# The societal cost of modifiable risk factors in Singapore

**DOI:** 10.1186/s12889-023-16198-2

**Published:** 2023-07-04

**Authors:** Vanessa Tan, Julian Lim, Katika Akksilp, Wai Leng Chow, Stefan Ma, Cynthia Chen

**Affiliations:** 1 Saw Swee Hock School of Public Health, National University of Singapore and National University Health System, 12 Science Drive 2, #09-01T, Singapore, 117549 Singapore; 2 Epidemiology & Disease Control Division, Ministry of Health, Singapore, Singapore; 3 Schaeffer Center for Health Policy and Economics, University of Southern California, Los Angeles, USA; 4 Department of Non-Communicable Disease Epidemiology, The London School of Hygiene & Tropical Medicine, London, UK

**Keywords:** Modifiable risk factors, Societal cost, Population attributable fraction, Global Burden of Disease

## Abstract

**Background:**

Singapore is one of the most rapidly ageing populations in the world. Nearly half of all disease burdens in Singapore are attributable to modifiable risk factors. This indicates that many illnesses are preventable by modifying behaviours such as increasing physical activity levels or maintaining a healthy diet. Prior cost-of-illness studies have estimated the cost of selected modifiable risk factors. However, no local study has compared costs between groups of modifiable risks. This study aims to estimate the societal cost attributable to a comprehensive list of modifiable risks in Singapore.

**Methods:**

Our study builds on the comparative risk assessment framework from the Global Burden of Disease (GBD) 2019 study. A top-down prevalence-based cost-of-illness approach was undertaken to estimate the societal cost of modifiable risks in 2019. These include healthcare costs from inpatient hospitalisation and productivity losses from absenteeism and premature mortality.

**Results:**

Metabolic risks had the highest total cost of US$1.62 billion (95% uncertainty interval [UI] US$1.51–1.84 billion), followed by lifestyle risks of US$1.40 billion (95% UI US$1.36—1.66 billion) and substance risks of US$1.15 billion (95% UI US$1.10—1.24 billion). Across the risk factors, the costs were driven by productivity losses, heavily skewed towards the older working-age group and among males. Most of the costs were driven by cardiovascular diseases.

**Conclusion:**

This study provides evidence of the high societal cost of modifiable risks and highlights the importance of developing holistic public health promotion programmes. As modifiable risks often do not occur in isolation, implementing effective population-based programmes targeting multiple modifiable risks has a strong potential to manage the cost of the rising disease burden in Singapore.

**Supplementary Information:**

The online version contains supplementary material available at 10.1186/s12889-023-16198-2.

## Introduction

Globally, nearly half of the total burden of disease and injury can be attributed to modifiable risk factors, such as low physical activity, dietary risks, and tobacco smoking [1]. In 2019, high blood pressure contributed to 10.8 million deaths worldwide and was the leading modifiable risk factor for death [2].

Modifiable risks account for high healthcare costs. Low physical activity costs healthcare systems US$53.8 billion worldwide [3]. Tobacco smoking was estimated to total US$422 billion in healthcare expenditure globally [4]. A recent study estimated that healthcare spending associated with 84 modifiable risk factors amounted to US$730 billion in the US [5]. In addition to substantial healthcare costs, modifiable risks also have a significant economic impact. A study on productivity losses in Poland found that premature mortality attributed to 20 modifiable risks amounted to US$5.3 billion [6]. Understanding the cost of modifiable risk factors can help to prioritise holistic public health programmes.

Modifiable risks rarely occur in isolation. Studies have shown that clustering risk factors typically have a synergistic effect [7]. This implies that the co-occurrence of multiple risk factors presents a greater likelihood of developing a disease than an individual risk factor alone. For instance, alcohol and tobacco smoking commonly occur together, and their combined effect leads to a greater risk of all-cause mortality [8]. High blood pressure also co-exists with high cholesterol, resulting in an elevated risk of cardiovascular disease [9]. Besides understanding the cost related to a single risk factor, estimating the aggregated cost generated by two or more risk factors in a population may be of greater interest as it can inform public health programmes to alleviate several health risks spontaneously.

In Singapore, healthcare spending has increased from S$3.8 billion in 2010 to S$11.1 billion in 2019 [10]. This was partly driven by an ageing population's more significant healthcare needs and the emergence of a more practical yet expensive medical technology [11]. Not accounting for COVID-19, healthcare spending is expected to increase to S$27 billion by 2030 and is likely to grow even more with the pandemic [12]. This places a significant toll on the healthcare system. Thus, measuring the societal costs can help prioritise healthcare policies by focusing on groups of modifiable risks with the highest societal cost. In Singapore, similar studies have been done to quantify the cost of smoking and a high-sodium diet separately [13, 14]. However, local research has yet to estimate the societal cost of groups of modifiable risks concurrently.

This study aims to fill the gap using the latest available data from the Global Burden of Disease Study (GBD) 2019 to estimate the societal cost attributable to modifiable risks in Singapore before the COVID-19 pandemic, which also represents the steady state of the healthcare system that is not interpreted by additional healthcare spending used for pandemic control and management. GBD is a multinational research study that quantifies the magnitude of ill health from diseases, injuries, and modifiable risk factors. Modifiable risk factors in GBD are identified through systematic reviews, census, and survey data. While the risk factors in GBD are by no means exhaustive, it is the most comprehensive as the risks and estimates are frequently updated to reflect changes in disease burden when new studies become available [2, 15, 16].

## Methods

### Study design

**Fig. 1:**
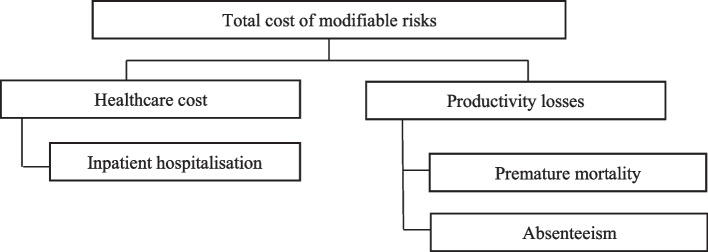
Components of the societal cost of modifiable risk factors

We estimated the societal cost of modifiable risks using a top-down prevalence-based cost-of-illness approach from the societal perspective. Cost-of-illness studies are often used to measure the costs attributed to a risk factor or health condition, considering direct and indirect costs [17]. In this study, direct costs refer to healthcare costs from inpatient hospitalisation, while indirect costs refer to productivity losses derived from absenteeism and premature mortality (Fig. 1). Data were obtained for individuals aged 20 years old and above. All estimates are reported in 2019 Singapore dollars (S$).

### Modifiable risk factors

Our study builds on the modifiable risk factors in the GBD 2019 study. The risk factors in GBD are organised into four nested levels. At the highest level, risk factors are grouped into three overarching risk factor categories: (1) behavioural risks, (2) metabolic risks, and (3) environmental and occupational risks. These are followed by more detailed risks nested within the preceding level. Behavioural risks contain nine Level 2 risks, metabolic risks contain six Level 2 risks and environmental and occupational risks contain five Level 2 risks. Certain Level 2 risks, such as occupational risks, consists of Level 3 risks which are further expanded into the specific type of exposure, such as to silica or sulfuric acid in Level 4. Not all Level 2 risks have detailed risks nested within them. Our study focuses on Level 2 risks, of which there are 20 risks as it reflects most of the modifiable risk factors.

To avoid confusion, we replaced "drug use", defined as dependence upon opioids, cannabis, cocaine, or amphetamines, with "illicit drug use", as these are prohibited in Singapore. We also replaced "maternal and child malnutrition" with "maternal malnutrition" to reflect the diseases related to the risk exposure in this study. This was because there were no episodes of inpatient admission that were classified or coded as child malnutrition.

Using Level 2 risk factors, we constructed five modifiable risk clusters, as shown in Table 1. First, risks within lifestyle and substance risks were identified through a systematic review of the evidence relating to the co-occurrence of multiple risk factors [18]. Risks within metabolic risks were identified based on the potential for similar policy interventions [19]. Risks not classified under these clusters were subsequently categorised into environmental and occupational risks or other modifiable risks.

**Table 1:** Risk factor clusters constructed using Level 2 risk factors in GBD 2019

Clusters	Level 2 Risk Factors from GBD 2019		
**Lifestyle risks**	High body mass index	Low physical activity	Dietary risks
**Substance risks**	Tobacco	Alcohol	Illicit drug use
**Metabolic risks**	High fasting plasma glucose	High LDL cholesterol	High systolic blood pressure
**Environmental and occupational risks**	Air pollution	Unsafe water	Occupational risks
	Other environmental risks	Non-optimal temperature	
**Other modifiable risks**	Kidney dysfunction	Childhood sexual abuse	Unsafe sex
	Maternal malnutrition	Intimate partner violence	Low bone mineral density

The risk factors in this study include only level 2 risk factors from GBD 2019. Some level 2 risk factors contain more detailed risks. For instance, lifestyle risks include high body mass index, low physical activity, and dietary risks. Within dietary risks, there are more detailed risks, such as a diet high in sodium and a diet low in fibre

### Estimation of Population Attributable Fraction (PAF)

The Population Attributable Fraction (PAF) is a key parameter used to estimate the attributable cost of diseases. PAF refers to the proportion of disease cases that can be averted if the risk factor exposure for a particular modifiable risk factor is reduced or eliminated [20]. For instance, although the significant cause of lung cancer can be associated with tobacco smoking, some can also be attributed to poor diet [21]. In this case, PAF can indicate the proportion of lung cancer cases that can be avoided without tobacco smoking.

However, there are challenges in estimating the PAF if the disease is associated with multiple risk factors. For instance, cardiovascular diseases can be attributed to several modifiable risk factors including but not limited to smoking, low physical activity levels, poor dietary risk, and high body mass index [22]. In such circumstances, because the disease can be prevented in multiple ways, the cases of individuals with numerous risks could be counted in the PAF several times. As a result, the PAF of individual risks can sum to more than 100% for a disease with multiple modifiable risks [23]. Thus, an approximation using an approach outlined by Krueger et al. was used to adjust for the overlap between risk factors in each cluster [24]. Other studies have also used this approach [25, 26].

### Data sources

Age- and sex-specific PAF and the number of deaths associated with each disease were extracted from the Global Health Data Exchange GBD Results Tool [27]. In GBD, a set of International Classification of Diseases 10^th^ Revision (ICD-10) codes are mapped to each disease. In addition, aggregated data on inpatient volume, inpatient costs and length of hospital stays by age groups (20–24, 25–29, …, 75–49, 80 and above), sex, and ICD-10 codes were extracted from the Singapore Ministry of Health administrative databases which cover all inpatient claims data from both public and private hospitals. In addition, the labour force participation rate and the median income were extracted from the Ministry of Manpower 2019 report [28].

### Estimating attributable healthcare cost

Healthcare costs were derived from inpatient costs. First, the attributable healthcare cost for each disease associated with the risk factor was computed by multiplying the corresponding cluster PAF with the disease-specific total inpatient costs. Next, the total inpatient cost for the disease was estimated using the disease-specific mean inpatient volume and bill. Finally, the attributable healthcare cost was added across all the associated diseases to give the attributable healthcare cost for the risk factor cluster. Costs were estimated separately for sex and age groups. The formula is presented in Table 2.

**Table 2:** Cost estimation formula for each risk factor cluster

Type	Formula
Healthcare Costs	$$\sum_{\mathrm{d}=1}^{{n}_{d}}\sum_{\mathrm{g}=1}^{2}\sum_{\mathrm{a}=1}^{{n}_{a}}{mean inpatient bill}_{\mathrm{d}ga}\times {inpatient volume}_{\mathrm{dga}}\times {\mathrm{Cluster }PAF}_{dga}$$
Productivity Loss due Absenteeism^1^	$$\sum_{d=1}^{{n}_{d}}\sum_{g=1}^{2}\sum_{a=1}^{{n}_{a}}{mean daily wages}_{ga}\times {LFPR}_{\mathrm{ga}}\times {\mathrm{mean LOS}}_{\mathrm{dga}}\times {inpatient volume}_{dga}\times {\mathrm{Cluster PAF}}_{\mathrm{dga}}$$
Productivity Loss due Premature Mortality^1^	$$\sum_{d=1}^{{n}_{d}}\sum_{g=1}^{2}\sum_{a=1}^{{n}_{a}}{number of deaths}_{\mathrm{g}a}\times {LFPR}_{\mathrm{g}a}\times {total expected future earning}_{\mathrm{g}a}\times {\mathrm{Cluster }PAF}_{d\mathrm{g}a}$$

where d = diseases; *n*_*d*_ = total number of diseases; g = sex; and a = age groups in 5 year interval; *n*_*a*_ = total number of age groups; PAF = population attributable fraction; LFPR = labour force participation rates; LOS = length of stay

^1^Productivity losses exclude those above 80

### Estimating attributable productivity losses

The human capital approach was used to estimate productivity losses [17]. This approach assumes that the value of future earnings is a proxy for future productivity. We included productivity costs for individuals aged 79 and below, accounting for the labour force participation rate. The formula used to estimate productivity losses from absenteeism and premature mortality is presented in Table 2.

Productivity loss from absenteeism refers to the income lost due to inpatient hospitalisation. This was computed by taking the product of the age- and sex-specific average daily wages with the mean length of stay, accounting for the cluster PAF, inpatient volume, and labour force participation rate.

Productivity loss from premature mortality refers to the value of human capital lost when a person dies prematurely. This accounts for the deaths attributed to the risk factor, estimated by taking the product of the number of deaths associated with the disease with the cluster PAF. The productivity loss from premature mortality was then estimated by multiplying the number of deaths attributed to the risk factor by the labour force participation rate and expected future earnings. Finally, the projected future earnings were approximated using the present value of lifetime earnings from death to age 79. The discount rate was set at 3%, and the income growth rate was designated as the annualised change in real wages in 2019 at 3.3% [29].

### Uncertainty

Uncertainty intervals (UI) were estimated by simulating the data using 1000 independent draws. The 95% UI was generated by the 2.5^th^ and 97.5^th^ percentiles. PAF was simulated from the beta distribution. The log-normal distribution was used to simulate data for inpatient costs, length of hospital stays, and the number of deaths. All analyses were done using R (version 4.0.5).

### Reporting

For reporting purposes, we categorised the age groups into three broad age groups (20 to 44, 45 to 64 and 65 and above, same as the US GBD Study) and the diseases into six aggregated categories (cardiovascular diseases, diabetes and kidney diseases, neoplasms, respiratory infections and tuberculosis, digestive diseases, and other diseases).

## Results

**Fig. 2:**
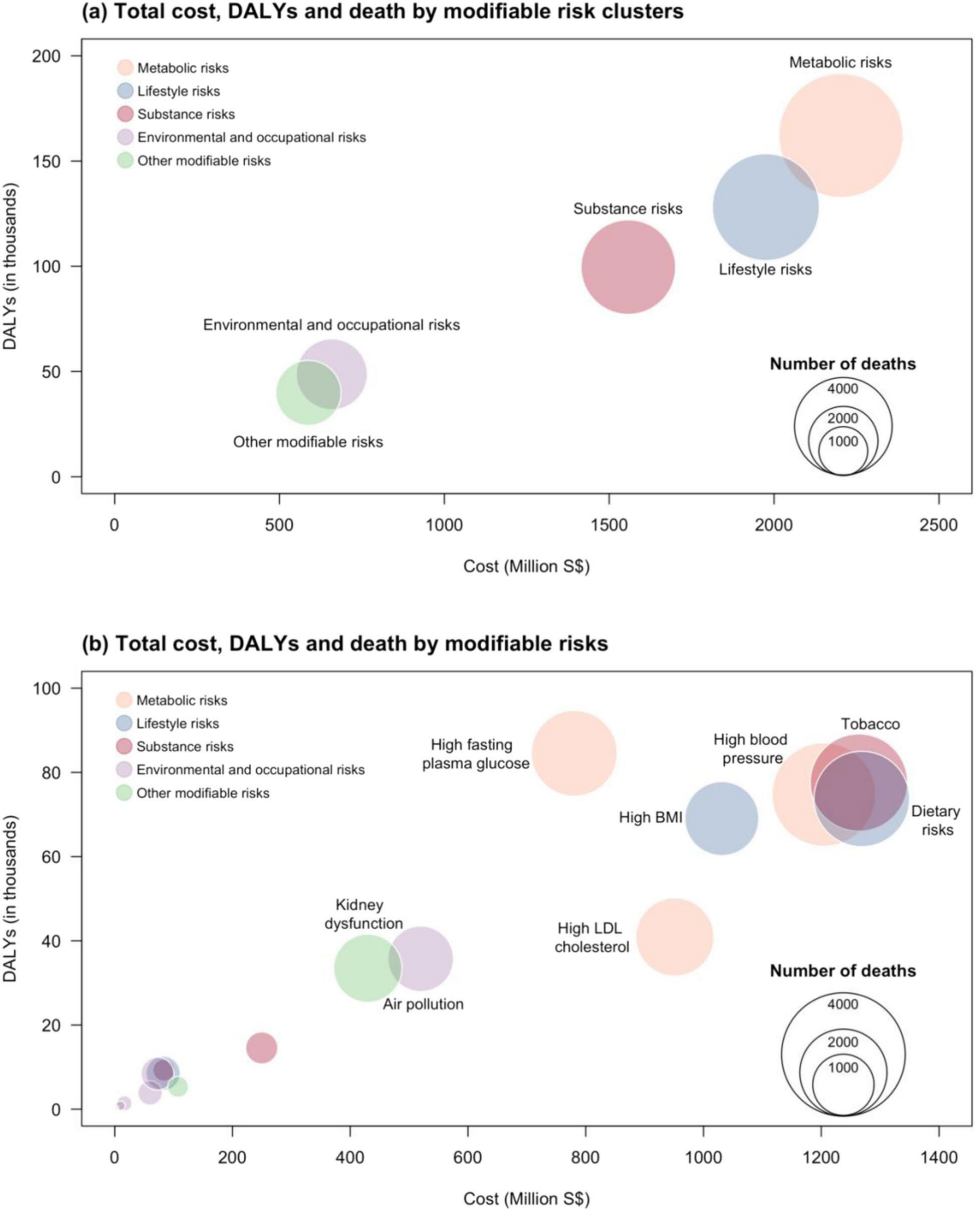
Bubble plot of the attributable total cost, disability-adjusted life years (DALYs) and death (**a**) by modifiable risk clusters (adjusted) and (**b**) by modifiable risks (unadjusted). The size of each bubble indicates the number of deaths attributable to the modifiable risks

At the cluster level, metabolic risks had the highest attributable cost and disease burden, followed by substance risks and lifestyle risks (Fig. 2A). At the individual risk level, the leading modifiable risk with the highest attributable cost and disease burden was tobacco smoking, followed closely by dietary risks and high blood pressure (Fig. 2B). Within dietary risks, a large proportion of the costs were from a diet high in sodium, diet low in whole grains and diet high in red meat (Supplementary Fig. 1).

### Total cost

The costs attributable to the risk factors are presented in Table 3. Metabolic risks had the highest total cost of S$2.20 billion (95% UI S$2.05 – 2.51 billion), followed by lifestyle risks of S$1.98 billion (95% UI S$1.85 – 2.26 billion) and substance risks of S$1.56 billion (95% UI S$1.49 – 1.69 billion). The lowest total costs were environmental and occupational risks (S$659 million, 95% UI S$610 – 759 million) and other modifiable risks ($588 million, 95% UI S$538 – 692 million).

**Table 3:** Breakdown of the attributable total cost

	**Cost, S$ in million (95% UI)**		
	**Total Cost**	**Healthcare Cost**	**Productivity Losses**
**Metabolic risks**	**2200 (2050, 2510)**	**642 (478, 909)**	**1560 (1530, 1630)**
High systolic blood pressure	929 (852, 1050)	218 (154, 321)	711 (672, 766)
High fasting plasma glucose	640 (554, 836)	271 (182, 451)	369 (350, 412)
High LDL cholesterol	633 (560, 741)	153 (94.5, 257)	480 (451, 514)
**Lifestyle risks**	**1980 (1850, 2260)**	**549 (417, 806)**	**1430 (1400, 1510)**
Dietary risks	1040 (952, 1200)	280 (198, 425)	759 (724, 809)
High body-mass index	868 (801, 995)	239 (186, 359)	629 (598, 686)
Low physical activity	68.1 (55.7, 111)	29.8 (20.2, 54.9)	38.3 (31.6, 60.6)
**Substance risks**	**1560 (1490, 1690)**	**333 (265, 443)**	**1230 (1210, 1280)**
Tobacco	1250 (1180, 1370)	286 (219, 395)	960 (938, 1010)
Alcohol use	234 (223, 261)	34.5 (26.4, 52.1)	199 (192, 216)
Illicit drug use	78.4 (71.9, 92.8)	12.6 (8.69, 20.0)	65.8 (61.1, 75.6)
**Environmental and occupational risks**	**659 (610, 759)**	**174 (130, 256)**	**486 (467, 532)**
Air pollution	506 (458, 588)	137 (98.3, 207)	368 (346, 407)
Occupational risks	68.4 (62.4, 80.0)	11.4 (7.39, 20.1)	57.8 (52.9, 64.5)
Other environmental risks	58.9 (53.8, 75.9)	14.8 (10.7, 24.8)	44.1 (41.2, 55.2)
Unsafe water, sanitation, and handwashing	16.3 (13.2, 24.0)	7.50 (4.55, 14.2)	8.83 (7.88, 10.5)
Non-optimal temperature	9.36 (7.85, 13.6)	2.74 (1.47, 5.49)	6.62 (5.85, 9.35)
**Other modifiable risks**	**588 (538, 692)**	**192 (141, 288)**	**398 (384, 425)**
Kidney dysfunction	430 (382, 522)	135 (89.3, 215)	295 (279, 317)
Unsafe sex	105 (97.1, 117)	14.3 (8.59, 23.8)	93.0 (87.5, 101)
Maternal malnutrition	45.8 (22.5, 94.3)	42.4 (19.8, 89.6)	3.37 (1.54, 7.48)
Intimate partner violence	3.22 (2.63, 3.96)	0.0142 (0.00862, 0.0312)	3.48 (2.89, 4.24)
Childhood sexual abuse	2.53 (1.79, 4.07)	0.493 (0.222, 1.15)	2.04 (1.47, 3.28)
Low bone mineral density	1.31 (1.24, 1.43)	0.0155 (0.00988, 0.0287)	1.30 (1.22, 1.41)

*UI* Uncertainty Intervals

Of the metabolic risks, high systolic blood pressure (S$929 million, 95% S$UI 852 – 1050 million) had the highest total cost, followed by high fasting plasma glucose (S$640 million, 95% UI S$554 – 836 million) and high LDL cholesterol (S$633 million, 95% UI S$560 – 741 million). Of lifestyle risks, dietary risks (S$1040 million, 95% UI S$952 – 1200 million) formed the highest total cost, followed by high body-mass index (S$868 million, 95% UI S$801 – 995 million) and low physical activity (S$68.1 million, 95% UI S$55.7 – 111 million).

Within substance behavioural risks, the total cost was mainly from tobacco (S$1250 million, 95% UI 1180 – $1370 million). Alcohol use accounted for S$234 million (95% UI S$223 – 261 million), and illicit drug use accounted for S$78.4 million (95% UI S$71.9 – 92.8 million). The highest attributable costs of environmental and occupational risks were air pollution (S$506 million, 95% UI S$458 – 588 million) and occupational risks (S$68.4 million, 95% UI S$62.4 – 80.0 million). Other modifiable risks that had substantial costs were kidney dysfunction at S$430 million (95% UI S$382 – 522 million) and unsafe sex at S$105 million (95% UI S$97.1 – 117 million).

### Total cost by disease category

**Fig. 3:**
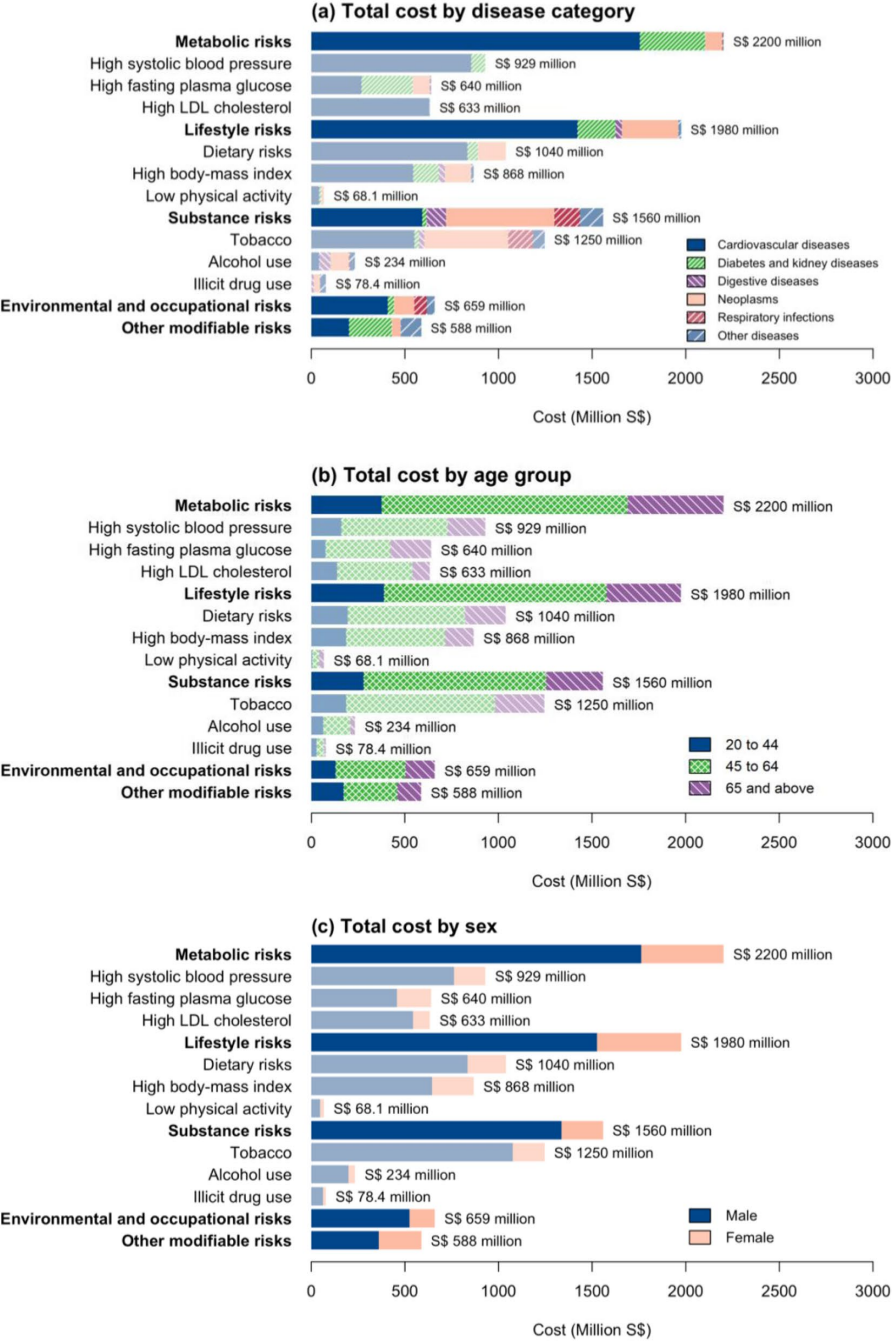
Attributable total cost of modifiable risk factors by (**a**) disease category, (**b**) aggregated age category and (**c**) sex

The total cost across the five risk clusters was primarily driven by non-communicable diseases such as cardiovascular diseases, diabetes and kidney diseases, and neoplasms (Fig. 3A**, **Table 4). Within metabolic risks, most of the costs were from cardiovascular diseases (S$1760 million, 95% UI S$1610 – 2020 million) and diabetes and kidney diseases (S$347 million, 95% UI S$254 – 521 million). Cardiovascular diseases accounted for nearly 80% of the total cost of metabolic risks. The costs within lifestyle risks were mainly from cardiovascular diseases (S$1420 million, 95% UI S$1310 – 1670 million), followed by neoplasms (S$302 million, 95% UI $286 - $357 million) and diabetes and kidney diseases (S$202 million, 95% UI S$148 – 322 million). For substance behavioural risks, most of the costs were driven by cardiovascular diseases (S$594 million, 95% UI S$536 – 675 million) and neoplasms (S$579 million, 95% UI S$556 – 630 million). Environmental and occupational risk costs were driven mainly by cardiovascular diseases (S$409 million, 9% UI 364 – 491 million). For other modifiable risks, the costs were driven by diabetes and kidney diseases (S$228 million, 95% UI S$193 – 296 million).

**Table 4:** Breakdown of total cost by disease category

	**Cost, S$ in million (95% UI)**		
	**Total Cost**	**Healthcare Cost**	**Productivity Losses**
**Metabolic risks**			
Cardiovascular diseases	1760 (1610, 2020)	1760 (1610, 2020)	1320 (1280, 1380)
Diabetes and kidney diseases	347 (254, 521)	183 (91.9, 357)	165 (154, 181)
Neoplasms	91.0 (81.1, 121)	20.3 (14.3, 33.8)	70.7 (62.9, 94.0)
Respiratory infections and tuberculosis	3.44 (2.74, 4.71)	1.13 (0.570, 2.22)	2.31 (1.96, 2.76)
Digestive diseases	0 (0, 0)	0 (0, 0)	0 (0, 0)
Other diseases	4.71 (3.39, 9.68)	0.99 (0.41, 2.53)	3.73 (2.66, 7.99)
**Lifestyle risks**			
Cardiovascular diseases	1420 (1310, 1670)	340 (223, 551)	1080 (1040, 1150)
Diabetes and kidney diseases	202 (148, 322)	106 (53.4, 217)	95.6 (87.4, 114)
Neoplasms	302 (286, 357)	68.4 (47.4, 107)	234 (228, 264)
Respiratory infections and tuberculosis	0 (0, 0)	0 (0, 0)	0 (0, 0)
Digestive diseases	34.6 (22.2, 53.0)	30.7 (18.4, 49.0)	3.93 (3.18, 5.23)
Other diseases	14.5 (12.6, 22.5)	4.25 (2.79, 8.24)	10.2 (9.0, 16.3)
**Substance risks**			
Cardiovascular diseases	594 (536, 675)	136 (86.0, 204)	458 (435, 489)
Diabetes and kidney diseases	22.5 (12.7, 44.1)	15.2 (5.83, 36.5)	7.33 (6.15, 10.1)
Neoplasms	579 (556, 630)	84.2 (62.6, 119)	495 (483, 527)
Respiratory infections and tuberculosis	137 (115, 200)	35.4 (16.5, 94.1)	102 (92.6, 114)
Digestive diseases	102 (87.2, 133)	38.4 (25.3, 63.8)	64.1 (56.2, 74.3)
Other diseases	123 (115, 145)	23.8 (16.1, 40.9)	99.5 (95.1, 111)
**Environmental and occupational risks**			
Cardiovascular diseases	409 (364, 491)	104 (68.0, 168)	306 (282, 346)
Diabetes and kidney diseases	32.4 (20.3, 66)	22.1 (10.0, 55.3)	10.3 (8.9, 13.1)
Neoplasms	108 (99.1, 126)	14.7 (8.44, 28.1)	94.5 (87.0, 106)
Respiratory infections and tuberculosis	66.8 (53.5, 110)	22.5 (9.2, 62.9)	44.3 (39.5, 52.5)
Digestive diseases	0 (0, 0)	0 (0, 0)	0 (0, 0)
Other diseases	41.9 (37.9, 52.2)	10.9 (6.85, 19.0)	31.0 (29.3, 35.2)
**Other modifiable risks**			
Cardiovascular diseases	202 (173, 252)	67.8 (42.7, 117)	134 (123, 150)
Diabetes and kidney diseases	228 (193, 296)	67.4 (32.9, 132)	160 (153, 172)
Neoplasms	48.0 (42.7, 56.1)	6.5 (3.1, 12.8)	41.5 (37.0, 47.5)
Respiratory infections and tuberculosis	0 (0, 0)	0 (0, 0)	0 (0, 0)
Digestive diseases	0 (0, 0)	0 (0, 0)	0 (0, 0)
Other diseases	110 (87, 161)	50.7 (26.9, 97.3)	61.8 (57.5, 68.1)

*UI* Uncertainty Intervals

### Total cost by age category

The total cost varied across age groups (Fig. 3B**, **Supplementary Table 1**)**. For metabolic risks, the older working-age group (aged 45 to 64 years) amounted to S$1.31 billion (95% UI S$1.19 – 1.55 billion), approximately 60% of the total cost. Conversely, the younger working-age group (aged 20 to 44) only accounted for S$377 million (95% UI S$355 – 422 million), 17% of the total cost. Across the risk clusters, most costs were from the older working-age group (aged 45 to 64 years).

### Total cost by sex

The total cost also varied by sex. Males had substantially higher attributable costs than females. For metabolic risks, males accounted for S$1.76 billion (95% UI S$1.61 – 2.04 billion), while females only accounted for S$440 million (95% UI S$373 – 583 million). This was true for all the clusters of modifiable risks (Fig. 3C**, **Supplementary Table 2).

### Healthcare cost

For healthcare costs, metabolic risks accounted for the highest costs (S$642 million, 95% UI S$478 – 909 million), followed by lifestyle risks (S$549 million, 95% UI S$417 – 806 million) and substance risks (S$333 million, 95% UI S$265 – 443 million) (Table 3). Most of the costs were from cardiovascular diseases. Across the risk factors, aged 45 to 64 years and 65 and above accounted for the most healthcare costs, while males accounted for most healthcare costs.

### Productivity losses

Productivity losses accounted for the largest proportion of the total cost. Metabolic risks had the highest productivity losses of S$1560 million (95% UI S$1530 – 1630 million), followed by lifestyle behavioural risks of S$1430 million (95% UI S$1400 – 1510 million) and substance behavioural risks of S$1230 million (95% UI 1210 – 1280 million) (Table 3). Attributable productivity losses were associated mainly with the older working age group and chronic conditions such as cardiovascular diseases and neoplasms.

## Discussion

This study is the first in Singapore to estimate the societal cost attributable to a comprehensive list of modifiable risks. Across the cluster of risks, metabolic risks have the highest cost in 2019, followed closely by lifestyle risks and substance risks. These costs were driven by non-communicable diseases, with cardiovascular diseases contributing to the highest cost.

Across the modifiable risks, the attributable costs were primarily driven by the older working-age groups (aged 45 to 64 years). However, many preventable diseases at an older age were found to be from long-term exposure to modifiable risk factors, implying that the risk may have existed at a younger age [30–32]. For example, a study reported that cumulative exposure to high body mass index during adolescence was associated with a threefold increase in cardiovascular diseases during adulthood [33]. As risk exposure accumulates from a younger age, it is essential for early and long-term health promotion programmes to be sustainable and target across the lifespan. Sustainable health promotion programmes to reduce risk exposure in the younger age group can lead to healthcare savings and productivity in the future.

We also found that most of the costs were concentrated among males, which could be primarily due to the higher labour force participation rate and income of men [28]. However, this gap may be narrowed down in the future with greater inclusion of women in the labour force and wage parity [34]. Nevertheless, productivity losses are expected to remain a significant driver of the societal costs, where public health policies and programmes should remain equitable for both sexes.

Relative to other modifiable risks, the proportion of disease burden attributable to metabolic risks was considerably higher. In addition, metabolic risks are prone to cluster together in an individual, resulting in an elevated risk of cardiovascular diseases [9]. In Singapore, where one in two people is projected to be above the age of 65 by 2050, the expected increase in the prevalence of metabolic risks and the associated increased risk of cardiovascular diseases is concerning as it is linked to higher healthcare costs. This highlights the importance of identifying those with metabolic risks so that prevention strategies can be targeted appropriately.

Lifestyle risks also contributed to considerable costs. Of the risks within lifestyle risks, the costs were notably driven by dietary risks, which comprise specific diets in 15 food types that are either under or over-consumed. The highest cost from dietary risk was from the diet high in sodium. A recent study in Singapore also reported high costs attributable to high sodium diet [13]. Despite efforts to reduce sodium intake in 2011, sodium consumption remained higher than the daily recommended amount even in recent years, highlighting the need for a timely review of existing policies [13, 35].

Even though substance risks are highly regulated in Singapore, these risks were also found to account for a sizeable cost. Tobacco smoking was estimated at S$1.29 billion, the highest cost out of all the individual risk factors. Existing policies have reduced smoking prevalence in Singapore from 13.9% in 2010 to 10.1% in 2020 [36]. These policies should be reviewed regularly as the tobacco industry evolves. Reducing tobacco smoking has a significant potential to reduce the high healthcare cost and economic burden on society.

Our study found that a large proportion of the societal cost stems from productivity losses, which also significantly affects employers. In addition to bearing the cost of lost productivity, employers are also subjected to higher insurance premiums the following year. Considering adults' time in the workplace, it offers an ideal setting to support health policies. Workplace interventions to promote a healthy lifestyle have been shown to reduce absenteeism and improve health outcomes [37]. The individual also plays a vital role in maintaining or improving their health, and public health efforts should empower individuals to be accountable for their health. Public health interventions should rely on more than just solid government support but also the collective effort of society.

To the best of our knowledge, this is the first study that estimates the societal costs of modifiable risk clusters. For example, a recent US study on healthcare spending attributable to modifiable risks finds that the highest spending was from high body mass index, followed by high systolic blood pressure, high fasting plasma glucose, and dietary risks [5]. Another study from Poland estimated the productivity losses from the same set of modifiable risks and found that the highest productivity losses were high systolic blood pressure, dietary risks, and high body mass index [6]. However, most studies on attributable costs focused only on a single risk factor. In contrast, the few studies that examined a comprehensive list of modifiable risks do not consider the co-occurrence of multiple risks in a cluster. Thus, these studies may be different due to methodological differences. However, while the studies in the US and Poland may not have estimated the cost by clusters, the hierarchy shows that the highest costs were from a combination of metabolic risks and lifestyle risks.

There are several limitations to our study. Firstly, our study shares the same methodological assumptions and constraints as the GBD study, which we extracted population attributable fraction (PAF) from. For instance, PAF estimates for risks such as occupational risks and childhood sexual abuse were based on sparse evidence and interpreting the cost estimates may require closer inspection [38]. Additionally, the PAF assumed reducing the risk factor to the theoretical minimum exposure level, which may not be a feasible public health goal in some cases. For instance, reducing smoking to its theoretical minimum of no tobacco use would require stringent guidelines and enforcement. Despite these issues, GBD is currently the most comprehensive study estimating health burdens relating to an extensive list of modifiable risk factors.

Secondly, the societal costs were underestimated in this study. Healthcare costs were derived only from inpatient hospitalisation. Other healthcare costs from primary care, and specialist outpatient clinics (SOC) were not included as they were not available at the time of this study. Given that many chronic diseases require routine chronic care management, more costs may have been incurred in primary care and SOC. However, the bulk of healthcare cost comes from the inpatient hospitalisation data [39–41]. In addition, productivity losses from absenteeism and premature mortality represent only a fraction of society's indirect cost. Productivity losses from presenteeism were not included, and productivity losses from absenteeism did not include sick days for routine check-ups or post-hospitalisation leave as these data were unavailable. Costs related to informal care were also not included. Informal care, provided by family members or friends, plays a significant role in the overall economic burden of a disease, especially for diseases which impairs activities of daily living. These costs include the time and effort devoted by caregivers, potential loss of income due to caregiving responsibilities, and other related expenses. Future research efforts could expand on the type of costs incurred.

Thirdly, risk factors between clusters are by no means mutually exclusive. In our study, we looked at the different modifiable risk clusters separately. However, other studies have also reported clustering in lifestyle risks and substance risks such as tobacco smoking, alcohol use, poor dietary risks, and low physical activity [42, 43]. Clustering patterns were also observed in metabolic and substance risks [44]. Future studies may consider the overlap of risk factor clusters.

Lastly, estimates were from 2019 and may not reflect the current burden. At the time of this report, the most recent PAF from the GBD study was available for 2019. Thus, healthcare spending and productivity losses could not be updated.

Nevertheless, these estimates are helpful as they reflect the societal costs before the pandemic, providing a baseline that can be used to assess the effectiveness of health programmes targeting modifiable risks. The government in singapore should prioritise programmes or interventions that seek to better control of metabolic risk (lowering systolic blood pressure, fasting plasma glucose, and LDL cholesterol), lifestyle risks (lowering dietary risks and body mass index), and substance risks (reducing tobacco and alcohol usage).This study offers evidence with significant policy implications. It is crucial to implement actionable plans and policies to reduce the growing disease burden. Identifying and quantifying the societal cost associated with modifiable risks equips policymakers with valuable insights to effectively target modifiable risks with a greater burden. Additionally, estimating the costs of modifying risk factors assists grantors in substantiating the cost-effectiveness of the funded programmes and interventions.

## Conclusion

This study shows that the burden of diseases from modifiable risks bare considerable costs to the individual, the healthcare system, and society. In 2019, metabolic risks accounted for the highest societal cost of S$2.20 billion (95% UI S$2.05 – 2.51 billion). Of the total cost for metabolic risks, the healthcare cost amounted to S$642 million (95% UI S$478 – 909 million), representing 5.6% of Singapore’s healthcare spending [10]. As the population ages, the disease burden attributable to the studied modifiable risk factors is expected to increase, along with healthcare costs and productivity losses. Implementing effective health programmes targeting multiple risks has a strong potential to manage the costs of the rising disease burden in Singapore and improve the health of the population.

### Supplementary Information

**Additional file 1**.

## Data Availability

The datasets analysed belong to the Ministry of Health Singapore. For confidentiality reasons, the datasets are not publicly available. Data are however available from the authors upon reasonable request and with permission of the Ministry of Health Singapore.
