# Correction to: The societal cost of modifiable risk factors in Singapore

**DOI:** 10.1186/s12889-023-16271-w

**Published:** 2023-10-16

**Authors:** Vanessa Tan, Julian Lim, Katika Akksilp, Wai Leng Chow, Stefan Ma, Cynthia Chen

**Affiliations:** 1 Saw Swee Hock School of Public Health, National University of Singapore, National University Health System, 12 Science Drive 2, Singapore, #09-01T, 117549 Singapore; 2 Epidemiology & Disease Control Division, Ministry of Health, Singapore, Singapore; 3 Schaeffer Center for Health Policy and Economics, University of Southern California, Los Angeles, USA; 4 Department of Non-Communicable Disease Epidemiology, The London School of Hygiene & Tropical Medicine, London, UK

**Corretion to: BMC Public Health (2023) 23:1285**

10.1186/s12889-023-16198-2

The original publication of this article contained an incorrect funding number. The incorrect and correct information is shown in this correction article, the original article has been updated.

**Incorrect:** NHG DSRB (reference number: 2020/00156)

**Correct:** NUS IRB (reference number: NUS-IRB-2021-611)

