# Supplementary material for: The societal cost of modifiable risk factors in Singapore
Source: BMC Public Health. 2023 Jul 4;23:1285. doi: 10.1186/s12889-023-16198-2 (PMC10318651; doi:10.1186/s12889-023-16198-2)
Supplement: Supplementary file 1 — Additional file 1. [file 12889_2023_16198_MOESM1_ESM.docx]

**Supplementary Figure 1**

Breakdown of the total cost for dietary risks by age group.


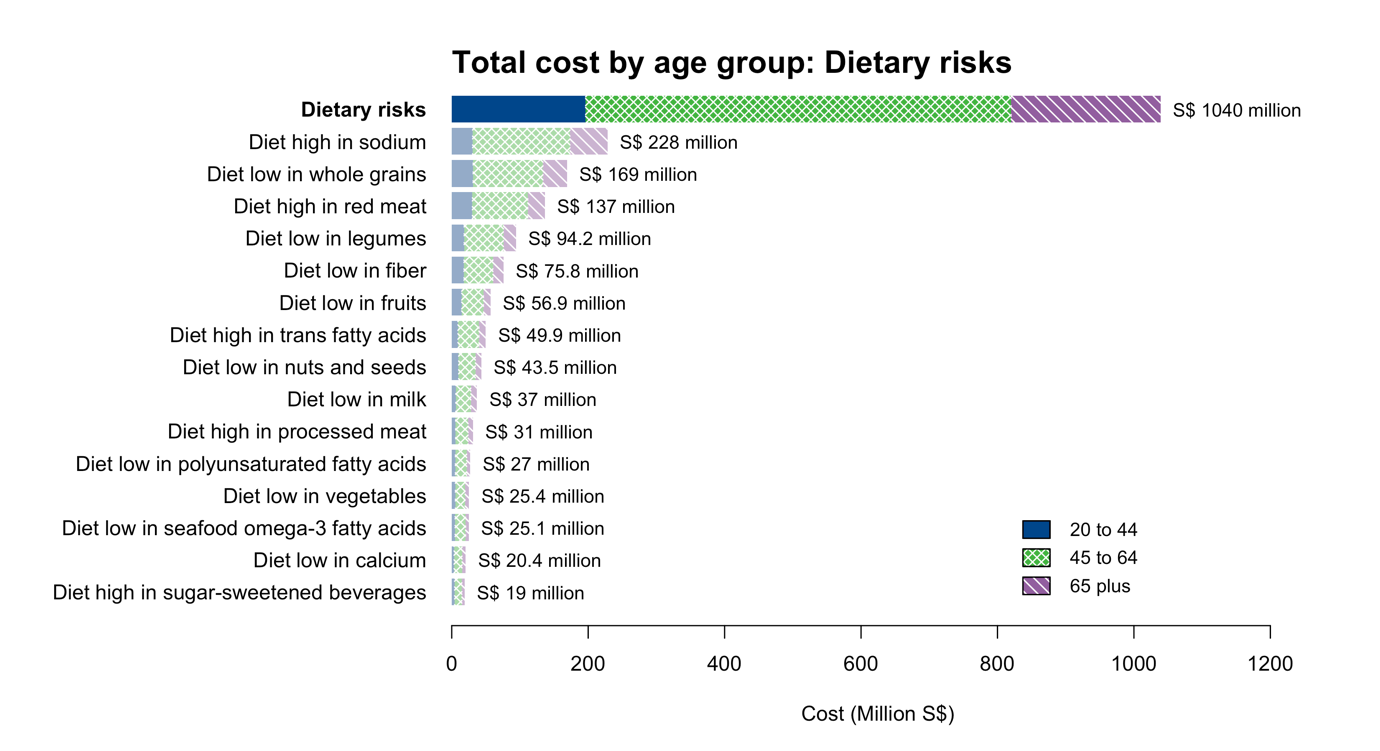


**Supplementary Table 1**

Breakdown of total costs by aggregated age category.

|  |  |  | | | | **Cost, S$ in million (95% UI)** | | | |  | | |
| --- | --- | --- | --- | --- | --- | --- | --- | --- | --- | --- | --- | --- |
|  |  | **Total Cost** | | | **Healthcare Cost** | | | | **Productivity Losses** | | | |
|  | | **20 to 44** | **45 to 64** | **65 and above** | **20 to 44** | | **45 to 64** | **65 and above** | **20 to 44** | | **45 to 64** | **65 and above** |
| **Metabolic risks** | | **377 (355, 422)** | **1310 (1190, 1550)** | **513 (401, 727)** | **40.8 (26.2, 72.1)** | | **290 (166, 519)** | **311 (198, 517)** | **337 (319, 367)** | | **1020 (989, 1080)** | **202 (191, 222)** |
|  | High systolic blood pressure | 161 (141, 188) | 567 (507, 653) | 201 (156, 279) | 12.6 (7.16, 24) | | 98.7 (57.9, 171) | 107 (66.6, 184) | 149 (132, 172) | | 468 (430, 511) | 94.2 (82.7, 106) |
|  | High fasting plasma glucose | 77.4 (66.2, 98) | 344 (294, 504) | 218 (156, 355) | 15.7 (9.09, 29.1) | | 107 (56.5, 244) | 149 (86.9, 285) | 61.7 (55, 73.8) | | 238 (221, 270) | 69.7 (59.1, 86.2) |
|  | High LDL cholesterol | 139 (123, 164) | 401 (344, 489) | 93.3 (62.4, 152) | 12.5 (5.7, 28.8) | | 84.8 (40, 170) | 55.2 (26.6, 111) | 126 (112, 143) | | 316 (293, 342) | 38 (31.6, 46.3) |
| **Lifestyle risks** | | **389 (366, 432)** | **1190 (1090, 1410)** | **399 (322, 557)** | **48.7 (33.7, 77.9)** | | **265 (171, 466)** | **236 (157, 391)** | **340 (323, 370)** | | **922 (889, 985)** | **163 (152, 182)** |
|  | Dietary risks | 196 (179, 224) | 624 (557, 753) | 219 (166, 326) | 20.2 (12.3, 36.1) | | 132 (77.4, 244) | 128 (78.8, 232) | 176 (162, 196) | | 492 (455, 533) | 90.8 (80.4, 104) |
|  | High body-mass index | 186 (167, 212) | 529 (481, 645) | 153 (123, 218) | 27.6 (19.9, 43.2) | | 124 (82, 208) | 88 (60.1, 149) | 159 (141, 179) | | 405 (376, 453) | 64.6 (57, 77) |
|  | Low physical activity | 6.39 (4.19, 13.1) | 34.2 (25.3, 62.3) | 27.5 (18.1, 49.2) | 0.876 (0.463, 2.27) | | 9.28 (5.21, 25.1) | 19.7 (10.7, 38.4) | 5.52 (3.58, 11.5) | | 24.9 (17.9, 44.3) | 7.85 (5.47, 13.7) |
| **Substance risks** | | **279 (267, 306)** | **975 (925, 1060)** | **303 (261, 382)** | **34.2 (24.3, 48.6)** | | **164 (116, 239)** | **135 (93.8, 207)** | **245 (236, 265)** | | **811 (789, 855)** | **169 (161, 181)** |
|  | Tobacco | 186 (173, 208) | 793 (738, 876) | 267 (226, 340) | 25.8 (17.2, 39.7) | | 141 (93.6, 216) | 119 (81.2, 191) | 161 (153, 175) | | 652 (626, 690) | 148 (140, 158) |
|  | Alcohol use | 64.3 (60.6, 72.7) | 144 (134, 164) | 25.6 (21.4, 34.3) | 6.13 (4.18, 9.68) | | 17.9 (11.4, 30.5) | 10.5 (6.85, 18.6) | 58.2 (55.5, 65.5) | | 126 (118, 139) | 15.1 (13.2, 18) |
|  | Illicit drug use | 28.7 (25.7, 33.6) | 38.7 (33.7, 48.7) | 11 (7.83, 17.4) | 2.3 (1.3, 4.03) | | 5.33 (3.09, 9.93) | 4.98 (2.69, 11.2) | 26.4 (23.7, 30.9) | | 33.4 (28.9, 41.4) | 6.01 (4.41, 8.36) |
| **Environmental and occupational risks** | | **130 (116, 154)** | **373 (337, 443)** | **156 (125, 225)** | **15 (9.84, 27.2)** | | **73.3 (44.8, 125)** | **85.5 (55.2, 148)** | **115 (104, 133)** | | **301 (281, 338)** | **70.2 (63.4, 80.5)** |
|  | Air pollution | 101 (86.9, 122) | 293 (255, 350) | 112 (84.9, 167) | 11.5 (6.95, 22) | | 61.3 (34.6, 107) | 64.6 (39.3, 119) | 89.4 (77.9, 105) | | 232 (211, 264) | 47.3 (41.2, 55.3) |
|  | Occupational risks | 11.6 (10.5, 13.4) | 35.5 (31, 42.7) | 21.3 (16.8, 29.3) | 1.12 (0.656, 2.05) | | 3.21 (1.76, 6.32) | 7.07 (3.6, 14.4) | 10.6 (9.59, 11.9) | | 32.9 (28.5, 38.6) | 14.2 (12, 17.5) |
|  | Other environmental risks | 11 (9.34, 15) | 33.8 (28.4, 46.4) | 14.1 (10.7, 21.6) | 0.938 (0.588, 1.9) | | 6.04 (3.4, 12.2) | 7.85 (4.76, 14.2) | 10 (8.6, 13.8) | | 27.7 (24.1, 36.5) | 6.27 (5.12, 8.81) |
|  | Unsafe water, sanitation, and handwashing | 3.7 (3.02, 5.1) | 6.46 (4.87, 8.82) | 6.16 (3.59, 12.5) | 1.18 (0.637, 2.35) | | 1.81 (0.76, 3.92) | 4.51 (1.89, 10.5) | 2.52 (2.18, 3.13) | | 4.66 (3.73, 5.89) | 1.65 (1.29, 2.27) |
|  | Non-optimal temperature | 2.48 (2.02, 4.12) | 4.61 (3.29, 7.22) | 2.27 (1.22, 4.63) | 0.231 (0.0929, 0.699) | | 1 (0.378, 2.5) | 1.51 (0.568, 3.79) | 2.25 (1.84, 3.72) | | 3.6 (2.68, 5.36) | 0.766 (0.471, 1.31) |
| **Other modifiable risks** | | **174 (149, 224)** | **286 (256, 337)** | **128 (93.4, 194)** | **58.8 (33.8, 109)** | | **56.3 (33, 97.8)** | **77.2 (41.8, 142)** | **117 (110, 128)** | | **229 (216, 250)** | **51.2 (46.7, 57.2)** |
|  | Kidney dysfunction | 72.8 (65, 86) | 234 (205, 284) | 123 (87.8, 189) | 10.2 (5.43, 19.9) | | 50.2 (27.4, 90.6) | 74.7 (39.5, 140) | 62.6 (56.9, 69.4) | | 184 (170, 203) | 48.3 (43.9, 54.3) |
|  | Unsafe sex | 50.7 (44.2, 59.8) | 49.2 (43.7, 56.4) | 5.19 (3.8, 8.08) | 6.27 (2.91, 14.1) | | 5.56 (2.63, 11.9) | 2.46 (1.14, 5.33) | 46.6 (42.1, 52.4) | | 43.6 (39.6, 49) | 2.79 (2.39, 3.35) |
|  | Maternal malnutrition | 45.5 (22.2, 94.2) | 0.244 (0.0459, 0.82) | 0 (0, 0) | 42.2 (19.8, 89.6) | | 0.21 (0.0211, 0.764) | 0 (0, 0) | 3.33 (1.51, 7.43) | | 0.0337 (0.0132, 0.0874) | 0 (0, 0) |
|  | Intimate partner violence | 2.65 (2.04, 3.38) | 0.543 (0.435, 0.683) | 0.0297 (0.022, 0.0402) | 0.00683 (0.00184, 0.024) | | 0.00517 (0.0038, 0.00692) | 0.00221 (0.00161, 0.00303) | 2.9 (2.31, 3.63) | | 0.538 (0.43, 0.678) | 0.0349 (0.0272, 0.0468) |
|  | Childhood sexual abuse | 1.2 (0.708, 2.19) | 1.24 (0.701, 2.43) | 0.09 (0.0313, 0.246) | 0.146 (0.0523, 0.407) | | 0.291 (0.0916, 0.869) | 0.0554 (0.0136, 0.184) | 1.05 (0.628, 1.99) | | 0.953 (0.542, 1.86) | 0.0345 (0.0126, 0.0896) |
|  | Low bone mineral density | 1.0 (0.927, 1.11) | 0.269 (0.246, 0.296) | 0.0422 (0.0366, 0.0512) | 0.00599 (0.00262, 0.0183) | | 0.00131 (0.000747, 0.00265) | 0.00815 (0.00455, 0.0154) | 0.998 (0.923, 1.1) | | 0.267 (0.245, 0.294) | 0.0340 (0.0304, 0.0389) |

UI: Uncertainty Interval

**Supplementary Table 2**

Breakdown of total cost by sex.

|  |  | **Cost, S$ in million (95% UI)** | | | | | | |
| --- | --- | --- | --- | --- | --- | --- | --- | --- |
|  |  | **Male** | | |  | **Female** | | |
|  | | **Total Cost** | **Healthcare Cost** | **Productivity Losses** |  | **Total Cost** | **Healthcare Cost** | **Productivity Losses** |
| **Metabolic risks** | | **1760 (1610, 2040)** | **442 (281, 695)** | **1320 (1280, 1380)** |  | **440 (373, 583)** | **200 (128, 342)** | **240 (233, 258)** |
|  | High systolic blood pressure | 762 (689, 865) | 155 (96.7, 240) | 608 (566, 657) |  | 167 (142, 215) | 63.5 (39.7, 110) | 104 (96, 117) |
|  | High fasting plasma glucose | 457 (391, 617) | 169 (103, 325) | 288 (266, 326) |  | 183 (141, 296) | 102 (57.4, 209) | 81.5 (77.6, 93.6) |
|  | High LDL cholesterol | 544 (472, 647) | 118 (62.2, 217) | 426 (396, 459) |  | 89.3 (71.6, 125) | 34.8 (18, 69.8) | 54.5 (49.9, 59.9) |
| **Lifestyle behavioural risks** | | **1530 (1410, 1790)** | **361 (235, 587)** | **1170 (1130, 1240)** |  | **448 (397, 557)** | **189 (136, 282)** | **260 (254, 278)** |
|  | Dietary risks | 835 (749, 979) | 199 (120, 332) | 636 (600, 684) |  | 204 (178, 261) | 81.7 (54.5, 131) | 123 (118, 133) |
|  | High body-mass index | 646 (581, 754) | 145 (101, 224) | 501 (468, 556) |  | 222 (194, 268) | 94.6 (67, 138) | 128 (122, 141) |
|  | Low physical activity | 46.3 (34.3, 79.8) | 17.2 (9.45, 37.8) | 29.1 (21.9, 50.1) |  | 21.7 (15.9, 37.1) | 12.6 (7.14, 26.3) | 9.14 (8.04, 12.5) |
| **Substance behavioural risks** | | **1340 (1270, 1460)** | **264 (197, 364)** | **1070 (1050, 1120)** |  | **222 (210, 255)** | **69 (54, 95.5)** | **153 (150, 166)** |
|  | Tobacco | 1080 (1010, 1190) | 229 (162, 330) | 847 (823, 888) |  | 170 (158, 199) | 56.8 (43.2, 83) | 113 (109, 123) |
|  | Alcohol use | 198 (187, 223) | 26.3 (18.4, 41.8) | 171 (163, 186) |  | 35.8 (32.8, 42.2) | 8.25 (5.64, 12.7) | 27.6 (25.9, 31.2) |
|  | Illicit drug use | 62.1 (56, 74.6) | 8.71 (5.36, 15) | 53.4 (48.7, 63.1) |  | 16.3 (14.2, 20.8) | 3.91 (2.29, 7.46) | 12.4 (11.2, 14.5) |
| **Environmental and occupational risks** | | **524 (481, 617)** | **119 (80.6, 195)** | **405 (383, 448)** |  | **135 (118, 178)** | **54.9 (38, 91.8)** | **81.1 (77, 89.7)** |
|  | Air pollution | 402 (357, 481) | 94 (61.3, 161) | 308 (283, 345) |  | 104 (88, 141) | 43.3 (28.4, 78.8) | 60.7 (56.5, 67.8) |
|  | Occupational risks | 56 (49.7, 66.9) | 8.47 (4.65, 16.7) | 47.6 (43.3, 54.4) |  | 12.4 (10.7, 15.4) | 2.92 (1.84, 4.87) | 10.2 (8.6, 11.7) |
|  | Other environmental risks | 48.6 (42.6, 62.7) | 10.9 (6.95, 19.5) | 37.7 (34.2, 47.6) |  | 10.3 (9.03, 14.9) | 3.94 (2.55, 7.54) | 6.34 (5.89, 8.24) |
|  | Unsafe water, sanitation, and handwashing | 10.7 (8.35, 16.3) | 3.96 (1.93, 8.76) | 6.74 (5.71, 8.24) |  | 5.63 (3.83, 10.2) | 3.54 (1.79, 8.03) | 2.09 (1.85, 2.52) |
|  | Non-optimal temperature | 6.39 (4.96, 10) | 1.54 (0.616, 3.78) | 4.84 (4.02, 7.11) |  | 2.98 (2.24, 4.96) | 1.2 (0.506, 2.7) | 1.78 (1.47, 2.72) |
| **Other modifiable risks** | | **361 (322, 436)** | **86.7 (52.2, 156)** | **274 (260, 297)** |  | **227 (194, 292)** | **106 (71.8, 170)** | **124 (118, 134)** |
|  | Kidney dysfunction | 308 (269, 383) | 85 (50.4, 155) | 223 (210, 245) |  | 122 (98.4, 166) | 50.1 (28, 93.7) | 71.4 (66.9, 77.2) |
|  | Unsafe sex | 49.4 (45.3, 54.2) | 1.28 (0.825, 2.05) | 48.1 (44.1, 53) |  | 55.7 (49, 67.1) | 13 (7.21, 22.7) | 45 (40.6, 51) |
|  | Maternal malnutrition | 0 (0, 0) | 0 (0, 0) | 0 (0, 0) |  | 45.8 (22.5, 94.3) | 42.4 (19.8, 89.6) | 3.37 (1.54, 7.48) |
|  | Intimate partner violence | 0 (0, 0) | 0 (0, 0) | 0 (0, 0) |  | 3.22 (2.63, 3.96) | 0.0142 (0.00862, 0.0312) | 3.48 (2.89, 4.24) |
|  | Childhood sexual abuse | 2.34 (1.57, 3.87) | 0.452 (0.183, 1.11) | 1.88 (1.28, 3.11) |  | 0.196 (0.126, 0.324) | 0.0408 (0.0194, 0.0901) | 0.155 (0.0987, 0.263) |
|  | Low bone mineral density | 0.626 (0.57, 0.692) | 0.012 (0.00677, 0.0245) | 0.614 (0.557, 0.677) |  | 0.689 (0.621, 0.773) | 0.00345 (0.00242, 0.00668) | 0.685 (0.618, 0.771) |

UI: Uncertainty interval
